# 2160 Does maternal schistosomiasis affect the humoral and cellular vaccine responses of infants?

**DOI:** 10.1017/cts.2018.67

**Published:** 2018-11-21

**Authors:** Deborah Bloch, Taryn McLaughlin, Cheryl Day, W. Evan Secor, Govert van Dam, Paul Corstjens, Heather B. Jaspan, Grace John-Stewart, Saad B. Omer, Lisa Cranmer

**Affiliations:** 1 Emory University; 2 United States Centers for Disease Control and Prevention (CDC); 3 Leiden University Medical Centre; 4 University of Washington

## Abstract

OBJECTIVES/SPECIFIC AIMS: The aims of this study are 2-fold: (1) to determine if maternal schistosomiasis affects maternal immunity to tetanus and/or transplacental transfer of antitetanus toxoid (TT) immunoglobulin G (IgG) from mother to infant and (2) determine the influence of maternal schistosomiasis on infant BCG vaccine immunogenicity. METHODS/STUDY POPULATION: The study will utilize blood samples from a historic cohort of 100 mother-infant pairs from Kisumu, Kenya, a schistosomiasis-endemic area. For the first aim, we will evaluate maternal schistosomal circulating anodic antigen, which has improved sensitivity and specificity to detect active schistosomiasis from serum, and antisoluble egg antigen IgG positivity compared with quantitative maternal anti-TT IgG at delivery and anti-TT IgG cord blood to maternal blood ratio (cord:maternal ratio). For the second aim, we will evaluate association between maternal schistosomiasis as detected by circulating anodic antigen and antisoluble egg antigen IgG at delivery and infant BCG-specific Th1-cytokine positive CD4+ cells at 10 weeks following BCG vaccination at birth. RESULTS/ANTICIPATED RESULTS: We hypothesize that active maternal schistosomiasis will be associated with decreased maternal anti-TT IgG and reduced efficiency of transplacental transfer, as measured by infant cord blood to maternal blood ratio of anti-TT IgG. We also expect that maternal schistosomiasis will be associated with decreased infant immunogenicity to BCG vaccine. DISCUSSION/SIGNIFICANCE OF IMPACT: This is a formative study on infant vaccine immunity using laboratory methodology not previously applied. Understanding infant immunity in the setting of maternal schistosomiasis will inform vaccination strategies and tailor vaccine development in schistosome-endemic areas such as Kenya, where neither TB nor neonatal tetanus have been eradicated. Additionally, our results will inform public health policies to consider integration of antischistosomal agents in antenatal care.

